# Molecular Pathogenesis, Immunopathogenesis and Novel Therapeutic Strategy Against COVID-19

**DOI:** 10.3389/fmolb.2020.00196

**Published:** 2020-08-11

**Authors:** Swapan K. Chatterjee, Snigdha Saha, Maria Nilda M. Munoz

**Affiliations:** ^1^Molecular Pharma Pvt., Ltd., Kolkata, India; ^2^Cagayan State University, Tuguegarao City, Philippines; ^3^De La Salle University, Manila, Philippines

**Keywords:** SARS-CoV-2, COVID-19, TMPRSS2, cytokine storm, Mannose-binding lectin, innate immunity, immunomodulators

## Abstract

The coronavirus disease 2019 (COVID-19), is a highly contagious transmittable disease caused by a recently discovered coronavirus, pathogenic SARS-CoV-2. Followed by the emergence of highly pathogenic coronaviruses in 2003 SARS-CoV, in 2012 MERS-CoV, now in 2019 pathogenic SARS-CoV-2, is associated with a global “pandemic” situation. In humans, the effects of these viruses are correlated with viral pneumonia, severe respiratory tract infections. It is believed that interaction between angiotensin converting enzyme 2 (ACE2) cell receptor and viral Spike protein mediates the coronavirus entry into human respiratory epithelial cells and establishes the host tropism. ACE2 receptor is highly expressed in airway epithelial cells. Along with viral-receptor interaction, proteolytic cleavability of S protein has been considered as the determinant of disease severity. Several studies highlight the occurrence of impaired host immune response and expression of excessive inflammatory response especially cytokines against viral infection. The mechanisms of SARS-CoV-2 induced acute lung injury are still undefined; however, the term *cytokine storm* has now been recognized to be closely associated with COVID-19. The levels of inflammatory mediators from *cytokine storm* cause damage to the host cells. In particular, the proinflammatory cytokine IL-6 appears to be the key mediator in early phase of virus-receptor interaction; however, secreted IL-6 might not be representative of lung inflammation. Understanding the cellular, and molecular factors involved in immune dysregulation and the high virulence capacity of COVID-19 will help in potential targeted therapy against it. “Drug repurposing” and “molecular docking analysis” is considered as an attractive alternative approach in analyzing suitable drug candidates to combat SARS-CoV-2 infection. Globally, extensive research is in progress to discover a new vaccine for novel COVID-19. Moreover, our review mainly focuses on the most state-of-the-art therapeutic approach mediated by “Mannose-binding lectin (MBL).” One of the most significant molecules of innate immunity is MBL. It plays a major role in the activation of the complement system as an ante-antibody prior to the response of any particular antibody. Recombinant human MBL can be used as immunomodulators against SARS-CoV-2.

## Introduction

Various viral members belong to the Coronaviridae family (COVs) under the order Nidovirales are continually circulating among the human population and responsible for causing mild to acute respiratory disease (Corman et al., 2020; Hoffmann et al., 2020). COVs member coronaviruses belong to the subfamily Coronavirinae and can be classified into alpha-, beta-, gamma-, and delta-CoVs. CoVs family members contain a positive-sense, single-strand RNA genome, 26 to 32 kilobases in length. The extensive distribution and infectivity capacity of CoVs establish them as an important pathogen. Besides numerous avian hosts, various member of CoVs have been recognized in a range of mammals, like bats, mice, masked palm civets, dogs, camels, and cats (Guan et al., 2003) and responsible for disease related to hepatic, respiratory, gastrointestinal systems, and nervous system in humans (Lu et al., 2020).

A new virus called the 2019 novel coronavirus (an enveloped beta-coronavirus) is identified in December 2019 and associated with a *de novo* contagious respiratory disease which primarily transpired in Hubei province, China (Huang et al., 2020; Wang C. et al., 2020). Initially, the infection was emerged from the Huanan seafood market and is initiated due to animal contact. Consequently, the disease spread through human-human contact within China and later rapidly spread causing new public health crises worldwide (Chan et al., 2020; Zhu et al., 2020). In early March 2020, this disease was documented as “pandemic” by the World Health Organization (Cucinotta and Vanelli, 2020).

Virologists are still uncertain about exactly how COVID-19 spreads. Medical doctors and scientists tend to agree that COVID-19 is transmittable through the inhalation of droplets from a person who has viral infection. However, the droplets discharged from cough and sneeze is heavy enough that might not travel more than 1 m (Corman et al., 2019). Unlike airborne particles that are much smaller than droplets and can stay in the atmosphere for a longer time. According to WHO, COVID-19 is not airborne; however, several experts appear to disagree. Common disinfectants like sodium hypochlorite; hydrogen peroxide etc., can be used to destroy the virus (Kampf et al., 2020). As per current information, subsequent transmission of this infection is possible through stool and/or contamination of the water supply (Huang et al., 2020). Although in most people the disease severity is mild, but the clinical manifestation of COVID-19 mainly spans from an asymptomatic state to pneumonia, acute respiratory distress syndrome, followed by multi organ dysfunction. Fever, headache, cough, fatigue, sore throat, myalgia, breathlessness, and conjunctivitis (in some cases) are the common clinical characteristics of this disease (Huang et al., 2020; Singhal, 2020).

Several phylogenetic analysis suggest that bat is likely the animal origin of the SARS-CoV-2 as it is 89% genomically identical with bat SARS-like-CoVZXC21, 50% with MERS-CoV and about 82% identical with human SARS-CoV (Zhou P. et al., 2020). Genome sequencing data also suggest that pangolins also have approximately 85.5–92.4% conserved sequence to SARS-CoV-2 (Yang et al., 2015). Other studies also suggest that SARS-CoV-2 could be a recombinant virus of bat coronavirus and snake coronavirus. The truth behind the origin of SARS-CoV-2 is yet to be discovered (Xie and Chen, 2020). The spike (S) glycoprotein, envelope (E) protein, membrane (M) protein, and nucleocapsid (N) protein are four structural proteins of novel SARS-CoV-2. The most significant surface protein is spike glycoprotein which interferes in establishing the association between the human respiratory epithelial cells to the virus via cell surface membrane receptor angiotensin-converting enzyme 2 (ACE2) and finally establishes the host tropism (Li et al., 2003). In this review, we emphasize the pandemic potential as well as molecular and immunological events involved in the pathogenesis of novel SARS-CoV-2. As the spike protein of SARS-CoV-2 plays a major role in the disease spreading, we comprehensively summarize the structure of spike protein and their potential role in the mutation of the virus. We also concisely discuss the potential effective treatments as well as new therapeutic approaches to control the transmission of this COVID-19 pandemic.

## Molecular Aspect of COVID-19 Pathogenesis

### Structural Configuration of Spike Protein

SARS-CoV-2 are single-stranded RNA viruses characterized by two groups of proteins, namely – (A) Structural Proteins - such as Spike (S) proteins marking all coronaviruses and bind to receptors on the host cell; Nucleocapsid (N) that protects the genetic information of virus, Matrix (M), and Envelope (E); (B) Replicase Complex Encoding Non-structural Proteins, such as proteases (nsp3 and nsp5) and RdRp (nsp12; Zhou P. et al., 2020). This enzyme is an ideal target as it helps the virus to replicate, meaning the virus depends on protease. Recent studies have shown that SARS-CoV-2 share a similar type of genomic organization with other beta coronaviruses. Like others, it produces a ∼800 kDa polypeptide upon transcription of genome. The proteolytic processing is mediated by papain-like protease (PLpro) and 3-chymotrypsin-like protease (3CLpro) and produces various non-structural proteins required for replication of viral particles. It is then predicted that these proteolytic enzymes could serve as the probable target site for anti-coronaviruses inhibitors (Yang et al., 2015). Recently, SARS-CoV showed an open reading frame 3a protein; coded by one of the group specific genes, and showed no sequence similarity to other known structural coronavirus proteins. It interacts with the E and S proteins while the M protein is glycosylated in all coronaviruses. Thus, the close homology of the M protein glycosylation and its likeness to 3a protein could result to investigate the glycosylation of these two membrane proteins (Song et al., 2018).

Viral transmembrane spike (S) glycoprotein is a trimeric class I fusion protein promotes the SARS-CoV-2 entry into the cell and the main target of neutralizing antibodies upon infection. Previous studies have shown that the function of spike protein depends upon its cleavage by host proteolysis enzyme into the S1 and S2 subunit. The attachment of the virus was facilitated by the S1 subunit whereas the S2 subunit assists in the fusion of viral and human cell membrane. Additionally, both the N-terminal domain (NTD) and the C-terminal domain (CTD) of the S1 subunit are capable in function as a receptor-binding entity (Millet et al., 2012). Although the S1 CTD region is utilized by both SARS-CoV and MERS-CoV to identify the receptor [receptor binding domain (RBD)], the region responsible for SARS-CoV-2 S-protein-hACE2 interaction remains unknown. Complete genomic analysis of each monomer of S-protein has shown that it has 22 predicted N-linked glycosylation sites and 4 predicted O-linked glycosylation sites. Studies conducted with Cryo-electron microscopy (Cryo-EM) have shown that 14-16 N-glycans are present on 22 potential sites in S-protein which are responsible for proper protein folding and priming of the protein by host proteases. The glycosylation pattern of the S-protein is one of the major features as it acts as a possible site for mutation and also facilitates the coronavirus to evade both innate as well as adaptive immune responses (Song et al., 2018). A recent study suggests that prediction of SARS-CoV-2 spike glycoprotein structure, glycan shield pattern and pattern of glycosylation has great inference on understanding the viral camouflage as well as the outline of cell entry, and also facilitate the development of new small-molecule drugs, vaccines, antibodies, and screening of the human host targets (Song et al., 2018).

Sequence alignment data done by the Clustal-W has shown that the S2 domain region (aa570–aa1278) of SARS-CoV-2 share a 91% identity with SARS-CoV spike glycoproteins. Though in the S1 domain (aa01–aa550), it shows a larger sequence difference (∼55% identity), which is essential for host cell adhesion, target interaction, and tropism of virulence but it has a conserved domain for ternary folding residue. This finding suggests that SARS-CoV-2 also capable to interact with SARS-CoV host targets like ACE2, CD26, Ezrin, and cyclophilins (Vankadari and Wilce, 2020).

### ACE2 Receptor – Major Entry Site for SARS-CoV-2

Receptor recognition is one of the major steps in viral infection of host cells and viral infectivity and pathogenesis. SARS-CoV depends upon ACE2 (angiotensin-converting enzyme 2) receptor which is highly expressed in human epithelial, endothelial, renal, cardiovascular tissue, and lung parenchyma (Tikellis and Thomas, 2012). Human ACE2 is a type I integral metallocarboxypeptidase act as a key player in the Renin-Angiotensin system (RAS). ACE2 exhibits protective function in the cardiovascular system, and other organs and act as a major target site for the treatment of hypertension. ACE2 protein is more profusely expressed on the apical surface of polarized epithelial cells as well as well-differentiated cells and certain progenitor cells in the bronchi (Li et al., 2003). Expression of ACE2 receptor in progenitor cells of respiratory tract cells with hair-like projections called cilia serves for the coronavirus entry site in the human body. Well-differentiated epithelial cells expressing ACE2 are readily infected by coronavirus. The viral infection thus correlates with the cell differentiation condition, ACE2 receptors expression, and localization of membrane binding. However, to date, there are still unanswered inquiries remain regarding ACE2 expression in human epithelial cells and its modulatory role in coronavirus. Questions include the type of epithelial cells involved in disease, the polarity of ACE2 expression on epithelial cells, and whether the coronavirus infection is ACE2 dependent. Interestingly, appearances of ACE2 receptor density on the progenitor cell surface increased with age and are generally present higher in men than in women (Xie et al., 2006). A study by Wu et al. (2020), using computer modeling has shown the presence of identical 3-D structures in the receptor-binding domain of the spike proteins of both SARS-CoV-2 as well as SARS-CoV. Biochemical interaction studies and crystal structure analysis by Wan et al. have proved that SARS-CoV-2 receptor-binding domain (RBD) contain residue 394 (glutamine), which has sequence similarity with SARS-CoV residue 479, and both can be accepted by the human ACE2 receptor on the critical lysine 31 (Wan et al., 2020).

Angiotensin converting enzyme 2 is an entry receptor for SARS-CoV-2 and shows 76% amino acid sequence homology with the SARS-CoV-S. Structural configuration study shows that, ACE2 contains 17 amino acid N-terminal signal sequences and a 22 amino acid hydrophobic transmembrane sequence near the C-terminus. ACE2 also contains 43 amino acid cytoplasmic domain, a potential phosphorylation sites, eight cysteine residues, and seven potential Af-linked glycosylation sites (Jia, 2016). The viral spike (S) protein of SARS-CoV-2 binds to cellular receptor ACE2 in a similar way to SARS-CoV-1 but with a 10- to 20-fold higher binding affinity. These findings suggest that increased ACE2 expression might confer easier transmissibility and also increase the susceptibility of SARS-CoV-2 into the host cell (Bourgonje et al., 2020). Studies using angiotensin-II receptor blockers (ARB) and ACE-inhibitors (ACE-i) suggest that the upregulation of cellular ACE2 expression facilitates the binding of SARS-CoV-2 and associated with severe disease manifestation. This receptor recognition by viral cell leads to host cell entry of the virus in combination with S-protein priming by the host cell protease TMPRSS2. Downregulation of ACE2 expression by SARS-CoV-2 could decrease the angiotensin-II clearance and lead to aggravation of tissue damage. Identification of interaction site and the downstream signaling cascade of SARS-CoV-2 and ACE2 receptor in the human cells will help to design the antibody-based therapeutic strategy (Gue et al., 2020).

A study by Zhou P. et al. (2020) on a mouse model of SARS-CoV infection, demonstrated that overexpression of human ACE2 receptor are associated with the severity of the disease. He also suggested that in pulmonary tissue alveolar epithelial type II (AECII) cells express 83% of ACE2 receptor and provide a suitable site for viral invasion. Additionally, gene ontology enrichment analysis of AECII has demonstrated that increased expression of ACE2 is associated with high levels of various viral process-related genes, including viral life cycle, genome replication, assembly, and regulatory genes for viral processes, etc. (Zhou P. et al., 2020). These findings imply that the ACE2-expressing AECII is able to assist coronavirus replication in the lung. Like pulmonary tissue some extra-pulmonary tissues such as heart, kidney, endothelium, and intestine express the ACE2 receptor (Li et al., 2020d). Another observation also suggests that a state of insulin resistance and elevated plasma glucose levels are associated with increased expression of ACE2 in lung epithelial cells and act as the risk factor for morbidity and mortality in SARS-CoV-2 infected patients (Finucane and Davenport, 2020). According to a study by Huang et al. (2020) suggest that in addition to lung epithelial tissue SARS-CoV-2 might affect other tissues including male tissues like testis and seminal vesicles. He also reported that SARS-CoV-2 infection might be related to cardiac injury (Huang et al., 2020). A recent study by, Holshue et al. (2020) unveiled that stool from a SARS-CoV-2 infected patient was positive for SARS-CoV-2, which suggests that this virus might infect the gastrointestinal tract. Significantly, high expression of ACE2 on the luminal surface of intestinal epithelial cells acts as a co-receptor for amino acid absorption from food. Studies also suggest that intestine might act as a major entry site for SARS-CoV-2 infection and infected epithelium of gut might have significant inference on fecal-oral transmission and viral spread confinement (Xiao et al., 2020).

### Priming of Spike Protein and Onset of Disease

Entry of corona-virus into the cell depends upon the priming of S-proteins by host cell proteases which comprise the cleavage at the arginine-rich site (multi-basic) S1/S2 and the S20 sites. The efficient cleavage of S-protein along with cleavage site sequence establishes the zoonotic potential of coronavirus. Various studies have proved that SARS-CoV-2 infection initiation and spread of disease into the host cells mainly depends upon S protein priming by TMPRSS2 (Transmembrane protease serine type 2), the serine protease. In humans, epithelial tissues, especially those lining the upper airways, bronchi, and lower airways show extensive TMPRSS2 expression (Hoffmann et al., 2020). The protein sequence analysis demonstrates that TMPRSS2 is conserved, with 78% sequence identity in human whereas, in mouse embryos and adult tissues, *In situ* hybridization analyses divulge the presence of TMPRSS2 in the epithelial cell lining the urogenital, gastrointestinal, and bronchi and bronchioles of respiratory tracts. However, the exact physiological function of TMPRSS2 within lung epithelial cells is not clear but various data suggest that it helps in proteolytic cleavage of the epithelial sodium channel and regulate sodium currents (Iwata-Yoshikawa et al., 2019).

According to the study, TMPRSS2 belongs to the type II transmembrane serine protease family, and play a major role in the cleavage of hemagglutinin (HA) molecule in the influenza virus upon entry into human airway epithelial cells in human (Hatesuer et al., 2013). However, it can cleave glycoproteins (Spike protein) and stimulates it to induce the fusion of virus-host cell membrane at the cell surface which in turn assists virus entry into the host cell. Various studies demonstrate that certain TMPRSS2 variants expression increases the threat of disease severity in influenza A (H1N1) infection (Hatesuer et al., 2013). A study by Hoffmann et al. (2020) had demonstrated that in infected cells, precleavage at the S1/S2 site by Furin might encourage consequent TMPRSS2-dependent entry and spread of infection. A type I transmembrane protein, Furin is an activating protease that plays a critical role in fusion of viral membrane and viral entry within the host cell. Thus the role of anti-TMPRSS2 as active-site inhibitors or inhibition of furin dependent entry might help us to consider them as probable therapeutic targets for influenza viruses and also for coronaviruses (Hoffmann et al., 2020).

### Pathogenesis and Immunopathology of COVID-19

The S protein present in the membrane of SARS-CoV-2 has been considered as the most potent virus entry determinant into the host cell through the receptor ACE2. A study by Belouzard et al. (2009) revealed that a significant cleavage event takes place by proteolytic enzymes at position S20 of SARS-CoV-2 S protein by TMPRSS2, results in the fusion of membranes as well as viral infectivity mediated by the release of viral RNA. Other studies also revealed that entry of viral RNA into the host cell depends upon not only membrane fusion, but also on the clathrin-dependent, and/or clathrin-independent endocytosis (Wang et al., 2008). After released into the cytoplasm viral RNA genome is translated into two structural proteins and poly-proteins, which help in viral replication. Upon infection with SARS-CoV-2 genome host cell activates well-coordinated and rapid immune response, i.e., innate and adaptive immune response, which represents the first line of defense against the viral infection. In endosome, membrane specific pattern recognition receptors (PRR), like Toll-like receptor (TLR3, TLR8, TLR7, and TLR9) or the cytosolic RNA sensor, RIG-I/MDA5 or the secretory type PRR like Mannose-binding lectin (MBL) and C-reactive protein (CRP) can recognize viral RNA as pathogen-associated molecular patterns (PAMPs) (Perlman and Netland, 2009). Interferon (IFN) type I activate a potent innate immune response against viral infection and also induce effective adaptive immune response. This recognition initiate a complex signaling cascade by recruiting adaptor proteins like mitochondrial antiviral-signaling protein (MAVS), IFN-β (TRIF), and stimulator of interferon genes protein (STING) and activate downstream cascades molecules, like adaptor molecule MyD88. This interaction activates transcription factors like nuclear factor-κB (NF-κB) and interferon regulatory factor 3 (IRF3) and helps in nuclear translocation. In the nuclei, these transcription factors induce the production of type I Interferons (IFN-α/β) and a plethora of pro-inflammatory cytokines especially IL-6 (Li et al., 2020a). Thus, interactions between the host cell and virus fabricate an assorted set of first line defense against the virus at the entry site. Type I IFN mediated activation of JAK-STAT pathway; initiate the transcription of IFN-stimulated genes (ISGs) under the control of the IFN-stimulated response element (ISRE). Accumulation of type I IFN can suppress viral replication and act as an immune modulator to promote phagocytosis of antigens by macrophage, as well as NK cells mediated restriction of infected cells. Thus, blocking the production of IFNs or disorder of the JAK-STAT signaling pathway or altered expression of macrophages has a direct effect on the survival of the virus within the host cell (Zhou Z. et al., 2020).

**FIGURE 1:**
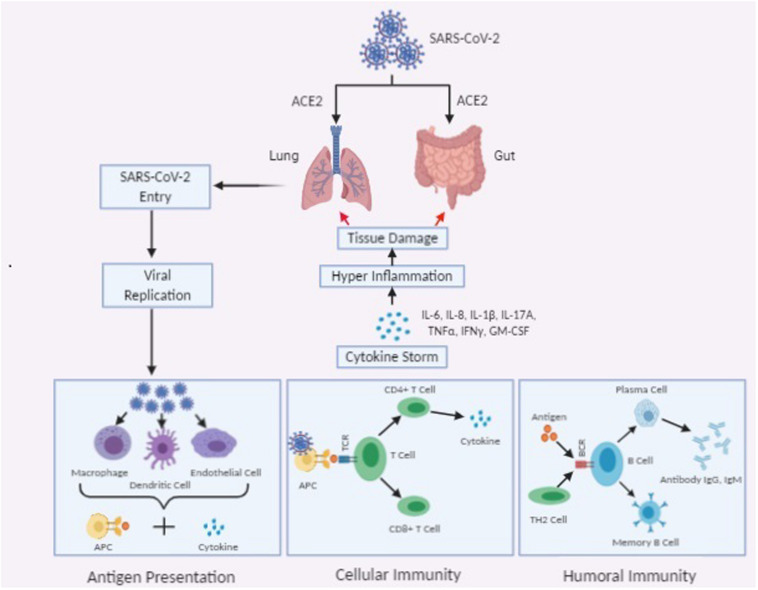
Schematic representation of immunopathogenesis of SARS-CoV-2 infection.

Generally, Th1 mediated immune response plays a predominant role in adaptive immunity against viral infections. T cell responses majorly depend upon the presence of APC (antigen presenting cells) mediated cytokine microenvironment. CD8^+^ cytotoxic T cells (CTLs) which are capable of secreting a cluster of molecules such as, granzymes, perforin, and IFN-γ are essential in the eradication of virus infected cells. CD4^+^ Helper T cells facilitate the overall adaptive response by assisting cytotoxic T cells. On the other hand, B-cell mediated humoral immune response, plays a protective role by producing the neutralizing antibody, and also impedes re-infection. According to some recent findings, in COVID-19 patients, an elevated level of chemokines and plasma cytokines like interleukins (IL-1, IL-2, IL-4, IL-7, IL-10, IL-12, IL-13, and IL-17), IP-10, macrophage colony-stimulating factor (MCSF), MCP-1, GCSF, hepatocyte growth factor (HGF), IFN-γ, MIP-1α, and TNF-α, etc., are associated with disease severity (Li et al., 2020e). Like in SARS and MERS the presence of “lymphopenia” and “*cytokine storm*” may have a significant role in the pathogenesis of COVID-19 (Moore and June, 2020). Moreover, like in cancer and other chronic infections, persistence of *cytokine storm* might stimulate necrosis or apoptosis of T cells, and leads to their exhaustion (Catanzaro et al., 2020). This “*cytokine storm*” is responsible for commencing of viral sepsis followed by lung injury induced by inflammation- which is related to other complications like acute respiratory distress syndrome (ARDS), pneumonitis, respiratory failure, sepsis shock, organ failure, and potentially death. Severity of COVID-19 in patients is associated with the marked decrease in the number of circulating B cells, CD8 + cells, CD4 + cells, natural killers (NK) cells, as well as a decrease in eosinophils, monocytes, and basophils (Zhou Z. et al., 2020; Figure 1).

## Molecular Docking, Prediction of New Drugs and Drug Repurposing

Novel drug development is a time consuming process that depends upon costly laboratory as well as clinical trials, possibilities of mistakes, and other conditions. So, now a day’s various bioinformatics tools like simulation, molecular docking, chemical stability studies, and target point determination playing an imperative and inventive approach in the designing of new drugs (de Ruyck et al., 2016). Among them “molecular docking” of any specific place provide a clearer idea about designing new drugs, examining and comparing their efficacy in any particular disease. In the case of “pandemic” caused by SARS-CoV-2 where there is no specific medicine or vaccine available, “drug repositioning” or “drug repurposing” plays an attractive role (Sheahan et al., 2020). However, such drugs require a clinical trial to show their effectiveness against the disease. In a recent study, Neda Shaghaghi used the crystal structure of SARS-CoV-2 proteinase and herbal medicines for docking analysis. According to the study terpenoids from the herbal medicines be able to impede the enzymatic cavity of important amino acids and inhibit the viral protease (Shaghaghi, 2020). Another study was done in virtual high throughput screening of clinically approved drugs and the structure of SARS-CoV-2 Mpro revealed that Saquinavir and Beclabuvir, Lopinavir, Ritonavir, and Nelfinavir act as the potential candidates for COVID-19 therapy (Sheahan et al., 2020). Molecular docking study is proved to be an economic method where technology-based ligand-protein interaction for a particularly active site reveals their possibilities as therapeutic candidates before synthesizing them. Various *in silico* studies based on molecular docking has proved US-FDA approved drugs like chloroquine, hydroxychloroquine, remdesivir, and arbidol as potential inhibitors of various viral polymerases and proteases and also known to be the most suitable targets for the SARS-CoV-2 treatment (Wang M. et al., 2020). Another interesting study on the SARS-CoV-2 spike glycoprotein and membrane CD26 docked complex model reveals a great interface among the proteins. This interaction between the loops of the S1 domain and the CD26 surface establishes CD26 as a potential binding site for S-protein along with ACE2 (Vankadari and Wilce, 2020). Studies were done on secondary metabolites of various Indian medicinal plants like garlic, curcumin, cardamom, ashwagandha, neem, aloe vera, harsingar, etc., have shown effective inhibition properties against SARS-CoV-2 protease enzyme (Srivastava et al., 2020). These compounds have an effective affinity toward the key amino acids active site and able to inhibit them during the catalytic process. Although the results obtained from drug repositioning or secondary metabolites of herbal plants by molecular docking provide information regarding suitable drugs candidate for COVID-19 therapy but thorough *in vitro* studies and *in vivo* studies can prove their effectiveness against SARS-CoV-2.

## Therapeutic Approaches Against COVID-19

Providing supportive care to the patients is the best strategy in current situations, as so far no established antiviral treatment is to be useful in controlling an outbreak of novel COVID-19 (Guo et al., 2020). So far numerous compounds have been checked in animal models or in cell culture to establish them as a potential therapeutic approach against entry or replication of SARS-CoV or MERS-CoV or SARS-CoV-2, but experiments done in animal or *in vitro* does not inevitably translate into efficacy in humans (Li and De Clercq, 2020). Based on the structural as well as the pathogenesis of COVID-19, continuous extensive investigations are in progress to determine the effective therapeutic agents for SARS-CoV-2 infections. Studies converge on drug discovery against emerging COVID-19 outbreaks can be broadly classified into two different modes of treatment: (A) virus-based therapy and (B) host-based treatment options.

### (A) Virus-Based Therapy

(i) Viral nucleic acids are made up of nucleosides and nucleotides. Drugs that are capable to target nucleotides or nucleosides and/or viral nucleic acids have a wide-range of activity against CoVs and other viruses. Studies have shown that various nucleoside analog, like Ribavirin, favipiravir, and galidesivir have antiviral activity against some animal CoVs, and in the SARS-CoV epidemic (Guo et al., 2020). (ii) Major enzymes and proteins involved in viral replication of COVID-19 are potential targets for anti-viral treatment. The PLpro enzymes and papain-like protease of SARS-CoV and MERS-CoV exhibit proteolytic, de-ubiquitylating as well as deISGylating activities. Studies have proven that among various protease inhibitors, lopinavir-ritonavir acts as the most effective one (Guo et al., 2020). (iii) The key immunogenic antigen of SARS-CoV-2 is membrane-anchored Spike glycoprotein, which plays an essential role in the host cell and virus interaction. Studies have shown that some monoclonal antibodies (mAbs) can target definite epitopes on the RBD of S1 subunit and restrain virus-cell receptor binding; on the other hand, others attach with the S2 subunit and disrupt virus-cell fusion (Jiang et al., 2014). Studies also suggest that mAbs exhibit neutralizing activities and also able to reduce viral titers *in vitro* as well as in small animal models. As S protein of SARS-CoV-2 displayed high homology to that of SARS-CoV, CR3022 the neutralizing antibody of SARS-CoV was found to bind potently with the RBD domain of SARS-CoV-2 (Tian et al., 2020). Previous studies also suggest that adoptive transfers of plasma which contain anti-MERS-CoV-S antibodies can block virus attachment and also accelerate viral clearance. Depending upon this idea, scientists are trying to develop convalescent plasma-based therapy against pandemic SARS-CoV-2 infection (Li et al., 2020c). Studies also imply antiviral peptides as a proposed analog for viral spike protein. Analogous peptides for the pre-transmembrane domain, N terminus, or the loop region which separates the HR1 and HR2 domains of SARS-CoV are competent to inhibit the formation of virus plaque (Tang et al., 2014). Some studies also prove that some siRNAs have antiviral activities *in vitro* but still they are in preclinical development (Wu et al., 2005). (iv) Along with E, M, and N proteins some accessory proteins are essential for virion assembly and viral replication by suppressing the host immune response. Anti-viral therapy against such proteins might have effective role in COVID-19 infection (Vigant et al., 2013). Studies have proved that LJ001 and JL103 (lipophilic thiazolidine derivatives) act as membrane-binding photosensitizers. They are proficient in the production of singlet oxygen molecules which stimulate various changes in lipid membranes results in the prevention of fusion between viral and host cell membranes (Zumla et al., 2016).

### (B) Host-Based Therapy

Host-directed therapies are related to the improvement of host immune response, host status, and/or handling of host-related factors coupled with viral replication. (i) Studies have shown that innate interferon response by host cells is important for viral replication within the host cell. Though CoVs are capable to restrain the interferon response to imply immune evasion, *in vitro* studies have revealed that treatment with various recombinant interferons might incline the infection severity (Menachery et al., 2014). (ii) To suppress dysfunctional systematic inflammation, Corticosteroids have excellent pharmacological effects. These are one of the major imunomodulators which were widely used in the treatment of SERS-CoV and MERS-CoV and are also considered as a helpful agent in the management of the current epidemic of SARS-CoV-2 (Li et al., 2020b). Another school of study suggests that combined use of both inducers of interferon along with innate immunomodulators might be efficient as antiviral agents against SERS-CoV-2. (iii) Apart from immunomodulators, other substances like metformin, atorvastatin, fibrates, as well as nutritional supplements might play the most important role in treating the current pandemic by boosting immunity (Zumla et al., 2016). (iv) CoVs generally utilize specific host factors like host receptors or other enzymes and proteins for viral entry as well as viral replication. Thus specific monoclonal or polyclonal antibodies, peptides, or functional inhibitors against host receptors or injecting an excessive liquid form of ACE2 receptor or recombinant technology must use to stop host membrane-viral fusion (Huentelman et al., 2004). (v) A recent study by Hoffmann et al. (2020) demonstrated that SARS-CoV-2 spike protein priming depends upon transmembrane protease serine 2 (TMPRSS2) for viral entry. Further studies also revealed that the serine protease inhibitor camostat mesylate, can block TMPRSS2 activity and considered as an attractive candidate for therapy (Hoffmann et al., 2020). (vi) Another study suggests that the entry of coronavirus into the host cell involved pH- and receptor-dependent endocytosis. A host kinase, AP-2-associated protein kinase 1 (AAK1) regulates clathrin-mediated endocytosis. Molecular docking studies reveal that the Janus kinase inhibitor baricitinib is a potent AAK1-inhibiting drug, is expected to be a suitable drug candidate for COVID-19 (Richardson et al., 2020; Table 1).

**TABLE 1:** Possible therapeutic approaches against SARS-CoV-2 infection.

Antiviral agent	Drug target	Mechanism of action	Infectious disease	References
**(a) Virus based treatment strategy**				
Ribavirin	RdRp	Inhibits viral RNA synthesis and mRNA capping	SARS-CoV-2, MERS-CoV, SARS-CoV, RSV, HCV	Guo et al., 2020
Favipiravir	RdRp	Inhibits RdRp	SARS-CoV-2, Influenza	Guo et al., 2020
Galidesivir	RdRp	Inhibits viral RNA polymerase function by terminating non-obligate RNA chain	SARS-CoV, MERS-CoV, IAV	Guo et al., 2020
Remdesivir	RdRp	Terminates the non-obligate chain	SARS-CoV-2, MERS-CoV, SARS-CoV	Guo et al., 2020
siRNA	RdRp	Short chains of dsRNA that interfere	SARS-CoV, MERS-CoV	Wu et al., 2005
Lopinavir	3CLpro	Inhibits 3CLpro	SARS-CoV-2, MERS-CoV, SARS-CoV, HCoV-229E, HIV, HPV	Guo et al., 2020
Ritonavir	3CLpro	Inhibits 3CLpro	SARS-CoV-2, MERS-CoV	Guo et al., 2020
Darunavir and cobicistat	3CLpro	Inhibits 3CLpro	SARS-CoV-2	Tian et al., 2020
CR3022	Spike glycoprotein	immunogenic antigen against Spike protein	SARS-CoV-2, SARS-CoV	Tian et al., 2020
Nafamostat	Spike glycoprotein	Inhibits spike-mediated membrane fusion A	SARS-CoV-2, MERS-CoV	Manli et al., 2020
Griffithsin	Spike glycoprotein	Griffithsin binds to the SARSCoV spike glycoprotein, thus inhibiting viral entry	SARS-CoV	Manli et al., 2020
Peptide (P9)	Spike glycoprotein	Inhibits spike protein-mediated cell-cell entry or fusion	Broad-spectrum (SARS-CoV, MERS-CoV, influenza)	Zhao et al., 2016
LJ001 and JL103	Lipid membrane	Membrane-binding photosensitizers that induce singlet oxygen modifications of specific phospholipids	Enveloped viruses (IAV, filoviruses, poxviruses, arenaviruses, bunyaviruses, paramyxoviruses, flaviviruses and HIV-1)	Zumla et al., 2016
**(b) Host-based treatment strategies**				
Recombinant interferons	Interferon response	Exogenous interferons	SARS-CoV-2, SARS-CoV, MERS-CoV	Menachery et al., 2014
Nitazoxanide	Interferon response	Induces the host innate immune response to produce interferons by the host’s fibroblasts and protein kinase R (PKR) activation	Coronaviruses, SARS-CoV-2	Manli et al., 2020
Chloroquine	Endosomal acidification	A lysosomatropic base that appears to disrupt intracellular trafficking and viral fusion events	SARS-CoV-2, SARS-CoV, MERS-CoV	Manli et al., 2020
Corticosteroids	Pulsed methylprednisolone	Patients with severe MERS who were treated with systemic corticosteroid with or without antivirals and interferons had no favorable response	SARS-CoV, MERS-CoV	Li et al., 2020b
Camostat Mesylate	Surface protease	TMPRSS2 inhibitor that blocks the TMPRSS2-mediated cell surface entry pathway	SARS-CoV, MERS-CoV, HCoV-229E	Hoffmann et al., 2020
Baricitinib	Clathrin-mediated endocytosis	AP-2-associated protein kinase 1 (AAK1) regulates clathrin-mediated endocytosis	SARS-CoV, SARS-CoV-2	Richardson et al., 2020
Convalescent plasma		Inhibits virus entry to the target cells	SARS-CoV, SARS-CoV-2, Influenza	Li et al., 2020c

*RdRp, RNA-dependent RNA polymerase; 3CLpro, 3C-like protease; IAV, influenza A virus; HCV, hepatitis C virus; RSV, respiratory syncytial virus; SARS-CoV, severe acute respiratory syndrome coronavirus; HCoV, human coronavirus; MERSCoV, Middle East respiratory syndrome coronavirus; and RBD, receptor-binding domain.*

### Glycosylation: A New Age Targeted Therapeutic Approach

**FIGURE 2:**
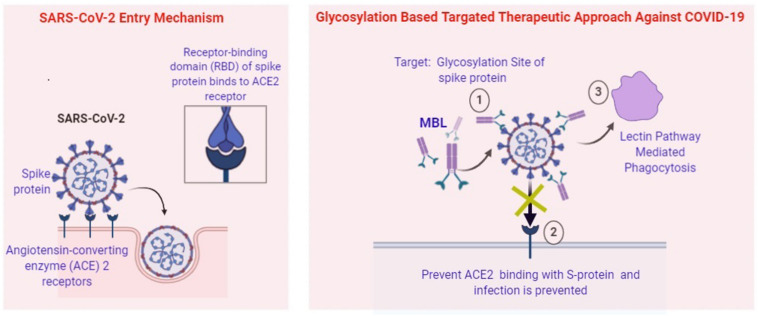
Potential Role of MBL in prevention of SARS-CoV-2. (1) Attachment of MBL at the glycosylation site of Spike Protein by “Lock and Key” mode. (2) Prevent ACE2 mediated entry of viral pathogen. (3) Lectin pathway-mediated phagocytosis of intracellular pathogens.

Carbohydrate-binding agents are considered as anti-CoV agents that target spike protein and restrain CoV entry. They are capable to bind specifically with the oligosaccharides present on the viral surfaces such as S and HIV glycoprotein (Garcia-Laorden et al., 2008). In *in vitro* condition as well as in mouse model they inhibit a wide range of CoVs, including HCoV-229E, SARS-CoV, HCoV-NL63, and HCoV-OC43. Glycans have a wide variety of shape, mass, charge, or other physical properties which help them to mediate extensive biological roles. Natural proteins called Lectins target the sugar moieties of a wide variety of glycoproteins which is not involved in cell adhesion. Most lectins belong to glycan families with distinct “carbohydrate-recognition domains” (CRDs) that conserve specific primary amino acid sequences or 3D structure and evolved from shared ancestral genes. In innate immunity, MBL acts as a key pattern-recognition molecule. It mainly functions as an ante-antibody, a humoral factor, crucial for the first-line host defense prior to the production of antibodies. Studies have shown that expression of MBL is associated with the initiation of complement activation via the lectin pathway and also responsible for opsonophagocytosis (Eisen, 2010). A study by Hartshorn et al. (1993) demonstrates that MBL function as an opsonin and inhibit hemagglutination as well as infectivity against respiratory viruses, such as influenza A virus. Binding of MBL with the N-linked high-mannose carbohydrate side chain present at the tip of S hemagglutinin protein is able to neutralize the infectivity of the influenza A virus. Studies also revealed that SARS-CoV S-protein contains 23 potential N-linked glycosylation sites. Binding of MBL with the S protein mannose side chains of SARS-CoV can be used as the most useful remedial agent (Ip et al., 2005). Thus, glycosylation of viral peptides might be considered as a novel therapeutic strategy against current COVID-19 pandemic (Figure 2).

## Discussion

Since, across the world, COVID-19 infection causes severe public health concern, analysis of the characteristics features of SARS-CoV-2, its interaction with the host receptor and immune responses, the phylogenetic and genomic similarity with other viruses will provide a clearer picture of diseases onset in individuals. Several groups of scientists have postulated that just like SARS-CoV, SARS-CoV-2 also depends upon the ACE2 as a receptor for host cell entry. The interaction of the virus transmembrane spike (S) glycoprotein with host cell receptors act as the determinant of the pathogenesis. A higher degree of disease severity is associated with viral load and age and sex of the patients. In elderly patients, the high viral load is associated with low immunity as well as higher expression of the ACE2 receptor in some hematopoietic cells, in cardiopulmonary tissues, and macrophages and monocytes. At the same time, male patients are more susceptible to the SARS-CoV-2 infection than their female counterparts. “Lymphopenia” (low blood lymphocyte count) is correlated with the clinical severity of COVID-19 related infection. However, lymphopenia can be considered as a biomarker of poor prognosis of COVID-19 which was also correlated with casualty in the influenza A (H1N1) pandemic in the year 2009. The study also, suggests that in one or more host species SARS-CoV-2 attached with integrins as cell receptors, through a conserved sequence RGD (403–405:Arg-Gly-Asp) with the RBD domain of the spike proteins. Pharmacotherapy against integrin can control the association between virus and integrin which is necessary to neutralize pathogenesis.

The development of SARS-CoV-2 infection majorly depends upon the interaction between the individual’s immune system and the virus. On one hand viral factors like virus type, mutation potential, virus viability along with the immune system factors of an individual like age, gender, nutritional status, genetics, neuroendocrine-immune regulation, etc., contribute to the severity of the disease. An effective immune response majorly depends upon a crucial part, called inflammation. Proper elimination of any infections majorly depends upon inflammation. Initial recognition of pathogens is the primary step for the onset of inflammatory response followed by the recruitment of immune cells. These immune cells help in the elimination of the pathogens and eventually lead to tissue repair and restoration of homeostasis. Though, in some infected individuals, SARS-CoV-2 induces extreme and prolonged cytokine/chemokine responses, called “*cytokine storm.*” This *cytokine storm* is related to multiple organ dysfunction or ARDS and later leads to physiological deterioration and death. An elevated level of serum cytokine IL-6 and C-reactive protein considered as the biomarker of severe β-coronavirus infection. Upon infection with β-coronavirus monocytes, macrophages, and dendritic cells get activated and start the secretion of prominent pro-inflammatory cytokine, like IL-6 along with other inflammatory cytokines. IL-6 can activate either classic *cis*-signaling or *trans*-signaling. Various pleiotropic effects on the acquired immune system (T and B cells) as well as on innate immune system [macrophages, neutrophils, and natural killer (NK) cells], are correlated with activation of *cis*-signaling and leads to cytokine release syndrome (CRS). On the other hand upon activation of *trans*-signaling, elevated IL-6 level creates the “*cytokine storm*” which is related with the secretion of monocyte chemoattractant protein-1 (MCP-1), vascular endothelial growth factor (VEGF), IL-8, and also excessive IL-6. It is also associated with reduced expression of adhesion molecule E-cadherin on endothelial cells. Reduced expression of E-cadherin and secreted VEGF mainly contribute to vascular permeability as well as leakage, which in turn coupled with the pathophysiology of hypotension and pulmonary dysfunction in acute respiratory distress syndrome (ARDS). An antagonist of IL-6 is tocilizumab, previously approved to treat juvenile idiopathic arthritis which is a rheumatic condition, is again “repurposed” for the COVID-19 pandemic. This finding suggests that we can potentially use IL-6 directed therapies not only in COVID-19 but also in other pandemics in the future involving Ebola and influenza viruses.

In this review, we summaries the different aspect of the therapeutic potential of the various anti-viral derivative. Finally, most reasonable options must be evaluated further in clinical trials against the COVID-19 pandemic. It might consist of either mono-therapy or combinational therapies comprise of interferon beta-1b, lopinavir-ritonavir, and/or mAbs and antiviral peptides. Thorough analyses of glycans are essential for the expansion of glycoprotein-based vaccine which might approach to correlate the immunogenicity with structural variations. In different expression systems, glycosylation act as a measure to evaluate antigen quality. Basic understanding correlated with RBD domain of the spike protein of SARS-CoV-2 consist of complex sialylated N-glycans and sialylated mucin type O-glycans will be useful to design suitable immunogens for vaccine development. MBL is a serum C-type lectin, which can bind SARS-CoV *per se* or infected cell and also capable to inhibit the infectivity of the virus. Studies have shown that “MBL-deficient” individuals are at more risk to SARS infection. We support, MBL as a potent therapeutic and prophylactic strategy in the prevention of SARS-CoV-2 pandemics. Shortly, it will be possible to develop broad-spectrum, novel, antiviral drugs active against a larger array of coronavirus, and also will be the ultimate treatment strategy for circulating and emerging COVID infections.

## Author Contributions

SC contributed to design, editing, and approval of final version of the manuscript. SS drafted and prepared the manuscript, and drew the figures. MM contributed to read and editing the draft of the manuscript. All authors contributed to the article and approved the submitted version.

## Conflict of Interest

SC and SS were employed by the company Molecular Pharma Pvt., Ltd., Kolkata, India. The remaining author declares that the research was conducted in the absence of any commercial or financial relationships that could be construed as a potential conflict of interest.
